# Strabismus and Artificial Intelligence App: Optimizing Diagnostic and Accuracy

**DOI:** 10.1167/tvst.10.7.22

**Published:** 2021-06-17

**Authors:** Laura Alves de Figueiredo, João Victor Pacheco Dias, Mariza Polati, Pedro Carlos Carricondo, Iara Debert

**Affiliations:** 1 Department of Strabismus, Hospital das Clínicas, University of Sao Paulo, Brazil; 2 IA para Médicos LTDA, Sao Paulo, Brazil; 3 Departament of Ophthalmology, Hospital das Clínicas, University of Sao Paulo, Brazil

**Keywords:** strabismus, artificial intelligence, smartphone app, eye version

## Abstract

**Purpose:**

Clinical evaluation of eye versions plays an important role in the diagnosis of special strabismus. Despite the importance of versions, they are not standardized in clinical practice because they are subjective. Assuming that objectivity confers accuracy, this research aims to create an artificial intelligence app that can classify the eye versions into nine positions of gaze.

**Methods:**

We analyzed photos of 110 strabismus patients from an outpatient clinic of a tertiary hospital at nine gazes. For each photo, the gaze was identified, and the corresponding version was rated by the same examiner during patient evaluation.

**Results:**

The images were standardized by using the OpenCV library in Python language, so that the patient's eyes were located and sent to a multilabel model through the Keras framework regardless of the photo orientation. Then, the model was trained for each combination of the following groupings: eyes (left, right), gaze (1 to 9), and version (−4 to 4). Resnet50 was used as the neural network architecture, and the Data Augmentation technique was applied. For quick inference via web browser, the SteamLit app framework was employed. For use in Mobiles, the finished model was exported for use in through the Tensorflow Lite converter.

**Conclusions:**

The results showed that the mobile app might be applied to complement evaluation of ocular motility based on objective classification of ocular versions. However, further exploratory research and validations are required.

**Translational Relevance:**

Apart from the traditional clinical practice method, professionals will be able to envisage an easy-to-apply support app, to increase diagnostic accuracy.

## Introduction

The list of health areas affected by digital transformation is long, and many analysts agree that technology and artificial intelligence systems will radically change medicine and reorient health care systems away from hospital and institutions to the home.^1^ Some analysts have even defined this as a new era of human progress.^2^

Artificial intelligence algorithms have been used to support the daily work of ophthalmologists. Certain common diseases, such as glaucoma, diabetic retinopathy, macular degeneration, corneal conditions and cataracts, are diagnosed and monitored on the basis of millions of data points provided by digital cameras.^3^

Ophthalmic instruments are constantly being improved and can now generate large volumes of complex diagnostic images. Although the data contained in these images exceeds human analytical resources, they have driven new artificial intelligence developments and applications.

In ophthalmology, fundoscopy and refractometric studies remain the main focus of the development of technologies.^4^ In the literature, studies have accurately identified and classified diabetic retinopathy,^5^^,^^6^ glaucoma,^7^ age-related macular degeneration,^8^^,^^9^ and retinopathy prematurity.^10^ Refractive errors can also be assessed from digital background photographs.^11^ As for evaluation of the patients with strabismus, the artificial intelligence has not yet been fully consolidated, especially in some semiological tests.

Clinical evaluation of eye versions plays an important role in strabismus semiological tests for diagnosis of special forms of strabismus associated with restricted eye movements. Eye versions are simultaneous movements of both eyes in the same direction.^12^ Versions can identify hypo or hyper muscle functions and incomitant eye movements.^13^

Most ophthalmologists evaluate eye versions by crude measures, by using the progressive scale with negative and positive numbers for hypofunction and hyperfunction, respectively.^14^ Despite the relevance of this evaluation, such measurements have low degree of reproducibility, because of examiner-dependent factors.

Quantitative measurements have been developed in an attempt to obtain a more objective scale. The Kestenbaum limb test^15^ and the lateral version light-reflex test are subjective assessments and subject to bias.^16^ Lim et al.^17^ advanced the version classification and quantified eye excursion in degrees in each of the cardinal positions, but they suggested selection bias in the choice of sample and limitations in accurate limbus measurement. Assuming that objectivity provides accuracy in clinical applicability, this study aims to develop an artificial intelligence tool that can assist the ophthalmologist's clinical practice, thus contributing to the semiological evaluation of ocular versions through images, making them less subjective, and, hence, more objective.

## Methods

### Study Design

The artificial intelligence application was developed by analyzing photographs showing how the eyes are positioned in the nine eye positions of gaze and the Python computer programming language. For this purpose, the tool was fed with images of the eyes in the nine positions of gaze, and their respective classifications were evaluated by the strabismus specialist. By using descriptive statistical analysis, the data were quantified from 1 to 9 with respect to the gaze and from −4 to 4 with respect to the version classification.

### Participants

The study was approved by the Research Ethics Committee of Hospital das Clínicas of the University of Sao Paulo. In total 323 patients with strabismus were invited to participate in the study. They had been followed up in a specialized outpatient clinic of a university hospital from 2015 to 2019. Patients who underwent orbit decompression or who had any facial deformity that prevented identification of facial points by the Dlib library were excluded. Patients with corneal disease such as microcornea and leukoma or patients for whom identifying the sclera corneal limbus region was difficult were also excluded. Other exclusion criteria included previous strabismus surgery or version classification less than or equal to −5 and greater than or equal to 5. The resulting sample had 110 participants, and their characteristics are shown in Table 1.

**Table 1.** Characteristics of the Study Participants

Total Patients	n = 110
Age (years)	
Medium age	41.47
IQR	45
Range	6–87
Sex	
Male	n = 52
Female	n = 58
Horizontal ocular deviation	
Exotropia	n = 42
Esotropia	n = 57

IQR, interquartile range.

The photographic images of the nine positions of gaze were obtained with the examiner standing in front of the participant, at 1 m, with a 16.1-million-megapixel digital camera (COOLPIX S8200; Nikon Inc., Tokyo, Japan) and ISO automatic gain (100–1600). The participant's head was positioned so that the head remained stable, and the patient looked at a fixation target corresponding to the position of gaze. All the images had resolution of all images was 4608 × 3456 pixels. This technique for obtaining and evaluating gaze versions by photography was validated by Lim et al.^17^

The patient was instructed to follow an object presented by the examiner, from the primary position to the secondary and tertiary positions of gaze. Each patient was evaluated twice by different evaluators present during the service. The evaluators were ophthalmologists who had been specialists in the area of strabismus for over a decade. In case of divergent evaluation, the Department Head, who had more than three decades of experience in the area, was consulted. For each muscle involved, versions were graded from −1 to −4 for hypofunction and from 1 to 4 for hyperfunction. The images of all the participants were classified into their nine positions, totalizing 990 images.

### Sample Treatment

Initially, the OpenCV library in Python language was used, initially, to standardize the images. Each eye was located, regardless of photo orientation (portrait or landscape), and a new square image cut out around them was generated. For this purpose, the image dimensions and colors were standardized, and the face inclination from the landmark facial extractor was corrected with 68 points obtained from the Dlib library, which provided its face center and generated an image cut for each eye.

 Then, the images were grouped on the basis of eye (left or right), gaze (1 to 9), and version (1 to 9), as shown in Table 2. The primary position of the image was not classified as a version, but it was only used as a reference gaze was classified as 5 and as a reference position.

**Table 2.** Reference Number of the Version and the Version Classification


1	Supradextro	−4
2	Supra	−3
3	Supralevo	−2
4	Dextro	−1
5	Primary position	0
6	Levo	1
7	Infradextro	2
8	Infra	3
9	Infralevo	4

The measurements of gaze excursion of the gaze and the subjective classification of the respective version were used to feed a Convolutional Neural Network that extracted the attributes of the images and their classification. Next, the angle of eye excursion of the eye from the primary position to the specified version was used in the process of ground truth labeling during Artificial Intelligence (AI) training.

ResNet50^18^ was used as the architecture. The ResNet50 network was imported directly through the Tensorflow Keras application module. This version was pre-trained on ImageNet, which has more than one million images in 1000 categories, allowing for a vast quantity of learned representations of these images, which were used for transfer learning. The last layers were removed, the remaining layers were frozen, and a new fully convolutional head was added to perform finetuning. The layers used were AveragePooling2D (7 × 7), Flatten, Dense (256), Dropout (50%), and Dense (with the number of classes of the gaze versions).

**Figure 1.**
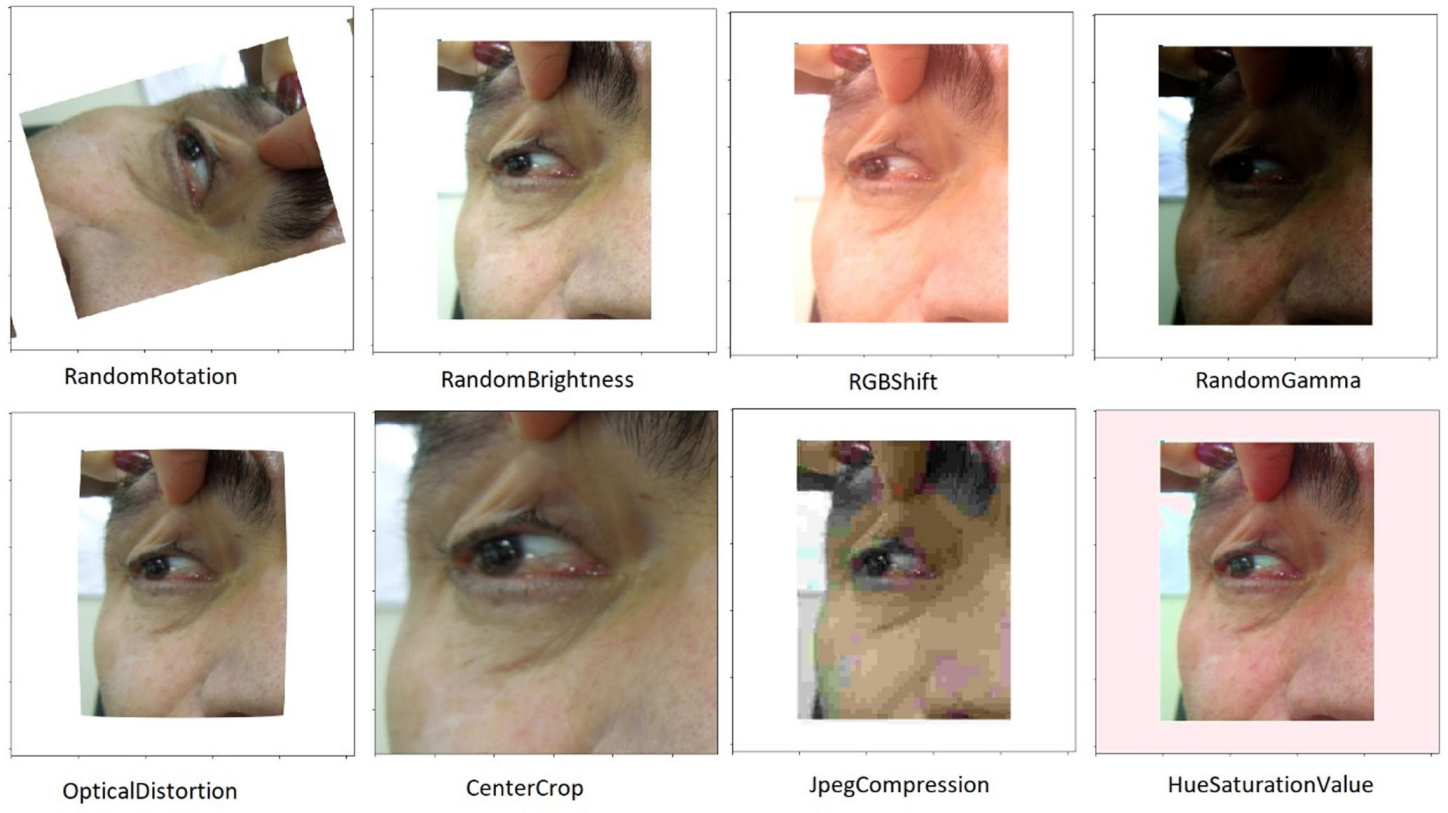
Example of the data augmentation technique for a patient from the study.

To increase the database size, the data augmentation technique was used. Through it, each image was selected at random and received some transformations, including up to five-degree rotation, up to 5% increase in width and height, up to 0.05 perspective distortion, up to 1% zoom, and up to 10% increase and decrease in brightness (Fig. 1). After this stage, the sample set consisted of more than 9600 images, which were separated into cross-validation sets for training, validation, and testing.

### The Convolution Neural Network

For the cross-validation process, the base was split into three parts: training, validation, and testing. In the first part, the model was trained to classify the eye versions correctly. In the sample set for validation, the model accuracy was measured according to the chosen metrics. After adjustments to the model, a final version was chosen and evaluated in a test set.

To train the neural network, 150 Epochs were used with a Learning Rate Finder and Batch Size function of 64 images. For quick inference via the browser, the StreamLit tool was used. The finished model was exported for use in mobiles through the Tensorflow Lite converter. For the transfer learning step on the pre-trained ResNet50, the last layers were removed, and the following layers were added: average pooling, flatten, dense, and dropout.

**Figure 2.**
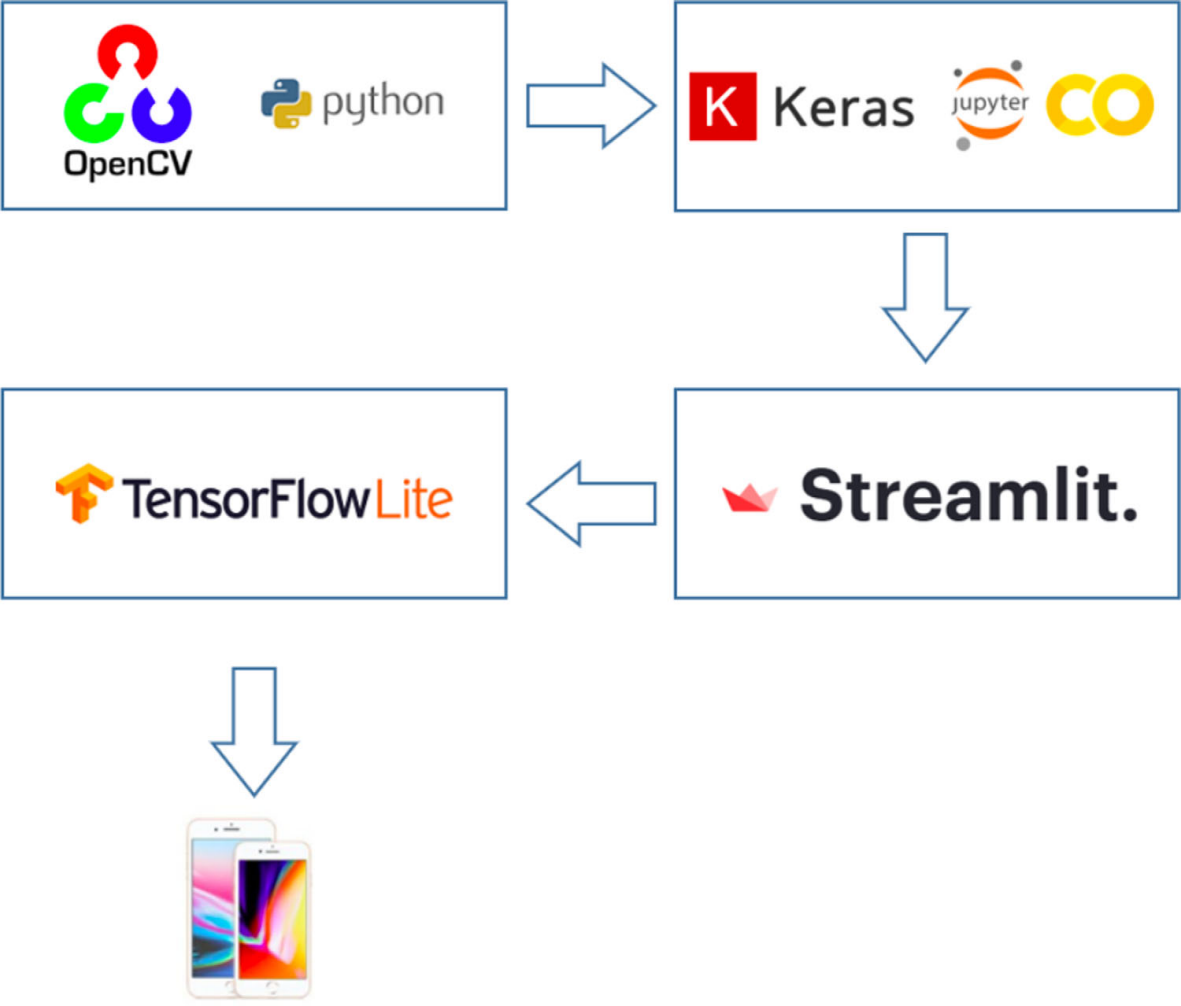
Flowchart of the platforms used during development of the application.

Figure 2 illustrates the flowchart of the sequence of platforms, from photo processing to conversion to Mobiles, used during development of the application. From the process of convolutional neural network creation, the mobile application could be created.

### The Version Estimation Application

**Figure 3.**
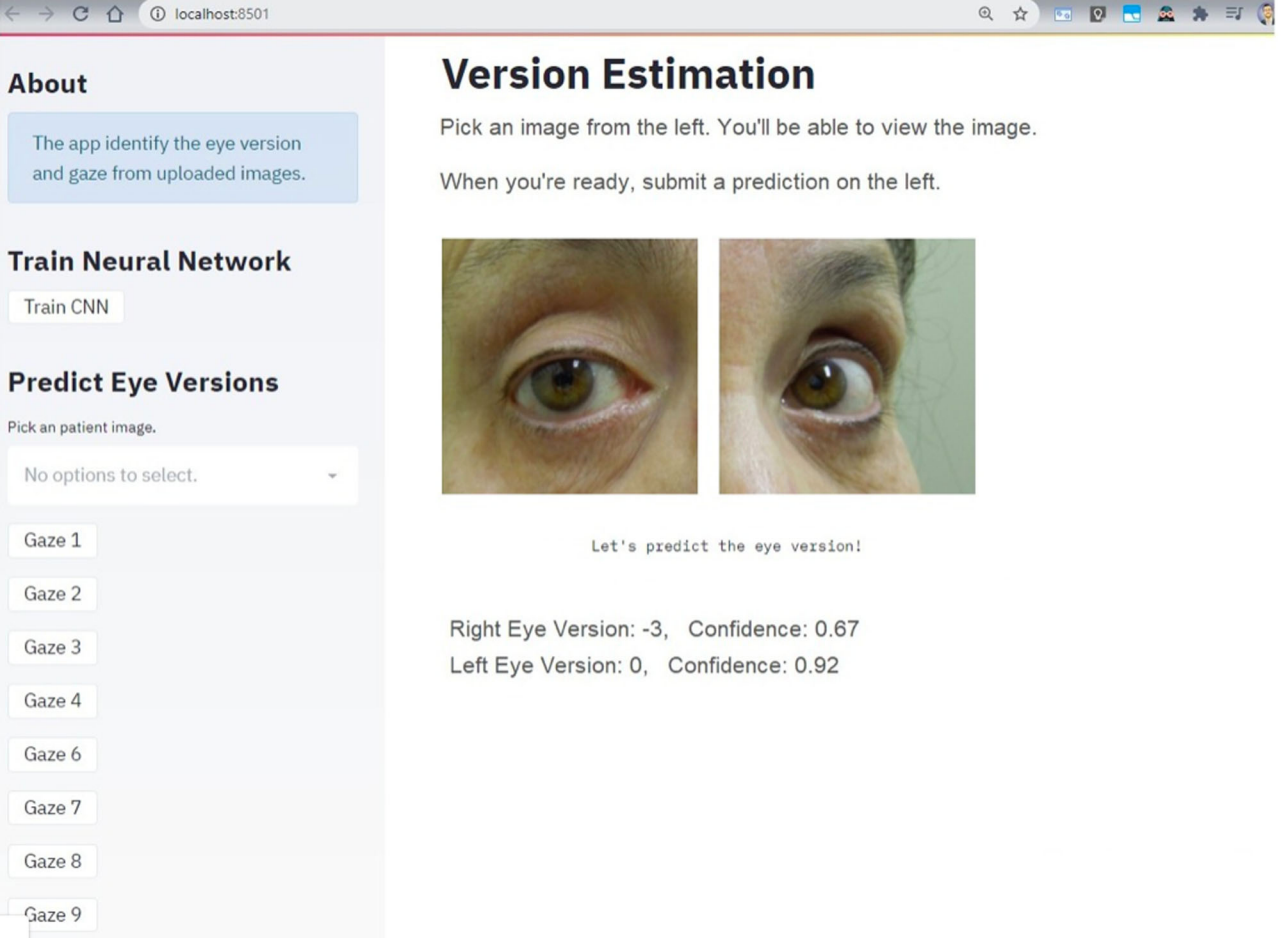
Application layout.

**Figure 4.**
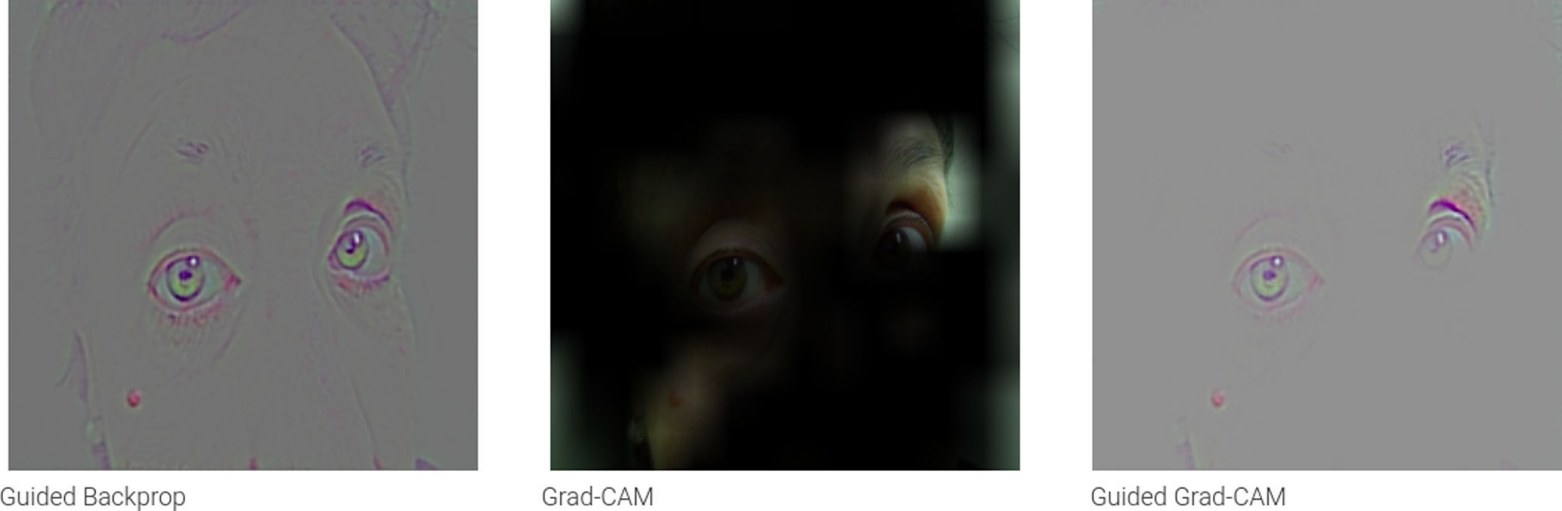
Gradient-weighted Class Activation Mapping (Grad-CAM) of an uploaded photo.

Figure 3 shows the final layout of the application and its functionality. The patient's photo showing both eyes at one specific gaze can be uploaded at the “Predict Eye Version” box, where there is also the “Gaze” dropdown menu to select. When the patient's photo is uploaded, the “Train CNN” box can be selected to predict the eye version. Then, the photo of the uploaded eyes appears separated above the text “Version Estimation,” and, above these photos, the predicted classification of the version in each eye and the corresponding confidence score are shown. Figure 4 depicts the Gradient-weighted Class Activation Mapping (Grad-CAM) generated by the uploading the image in Figure 3.

**Figure 5.**
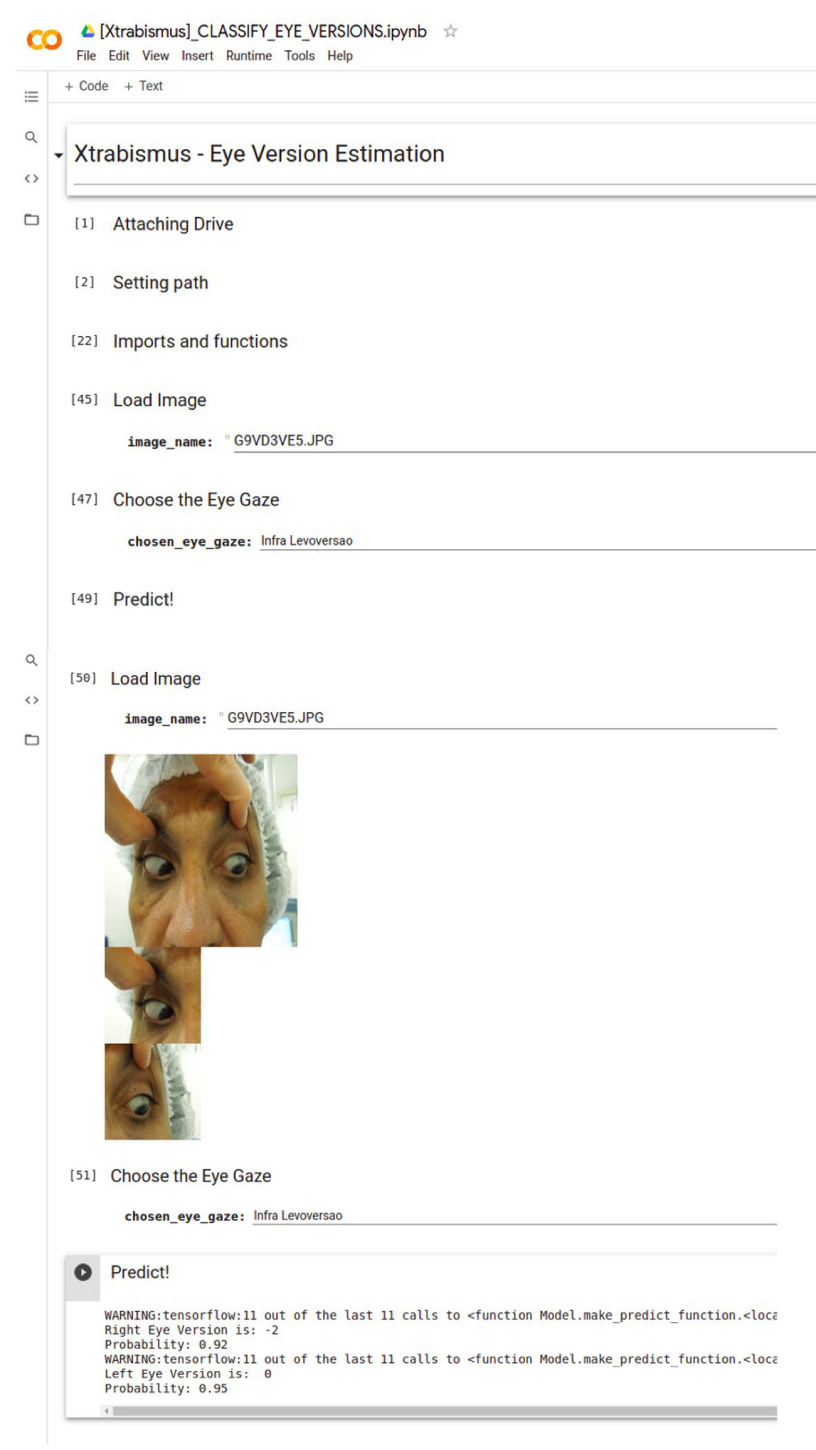
Application layout via Google Colab.

Although we used the Streamlit framework was used to create the app, a version via Google Colab was also generated to simplify the use by any professional without the need for complex installation and the same steps, as can be seen in the video in the Supplementary material. Figure 5 shows the App layout.

### Statistical Analysis

The classification performance measures recall, precision, F-score, and support were used in the sample to relate the eye position of the eye to its version. Recall, or sensitivity, is the proportion of true-positive cases that are correctly predicted positive. Precision denotes the proportion of predictive positive cases that are correctly true positives. The F-score is the weighted harmonic average of precision and recall. Support is the number of observations in which eye gaze and eye version are combined. For validation purposes, the model accuracy was measured according to the chosen metrics, and 15% of the images in each class were used.

## Results

Tables 3 and 4 detail the precision, recall, F1-score, and quantity of patient´s photo by eye, gaze, and version for the right and left eye, respectively. Table 4 does not show gaze 9 because there was no patient with this combination.

**Table 3.** Results for the Right Eye

Gaze	Version	Precision	Recall	F1-Score	Support
1	1	0.70	0.64	0.67	11
	2	0.33	0.92	0.49	12
	3	0.00	0.00	0.00	12
	4	0.40	0.50	0.44	4
	5	0.00	0.00	0.00	6
	6	0.00	0.00	0.00	1
	7	0.00	0.00	0.00	1
	9	0.00	0.00	0.00	1
2	1	0.53	1.00	0.69	9
	3	1.00	0.85	0.92	13
	5	0.00	0.00	0.00	6
3	3	1.00	0.11	0.20	9
	4	0.59	1.00	0.74	10
	5	0.20	1.00	0.33	1
	6	1.00	0.25	0.40	4
	8	1.00	1.00	1.00	1
4	2	0.57	1.00	0.73	4
	3	0.00	0.00	0.00	2
	5	1.00	0.00	0.80	5
6	1	0.27	0.75	0.40	4
	2	0.50	0.22	0.31	9
	3	0.54	0.78	0.64	9
	4	0.62	0.76	0.68	17
	5	1.00	0.50	0.67	2
	6	0.75	0.60	0.67	5
	7	0.00	0.00	0.00	7
	8	0.50	0.33	0.40	3
7	3	0.92	1.00	0.96	11
	4	0.00	0.00	0.00	1
8	1	0.43	1.00	0.60	3
	2	1.00	0.17	0.29	6
	3	0.55	1.00	0.71	6
	4	1.00	1.00	1.00	2
	5	0.00	0.00	0.00	2
	6	0.00	0.00	0.00	2

**Table 4.** Results for the Left Eye

Gaze	Version	Precision	Recall	F1-Score	Support
1	2	0.00	0.00	0.00	2
	3	0.32	1.00	0.48	6
	4	0.67	0.75	0.71	8
	5	0.67	0.17	0.27	12
	8	0.00	0.00	0.00	3
2	1	0.00	0.00	0.00	1
	3	0.77	1.00	0.87	10
	5	0.75	1.00	0.86	3
	6	0.00	0.00	0.00	3
3	1	0.56	1.00	0.71	10
	2	1.00	0.45	0.62	11
	5	0.00	0.00	0.00	1
	6	1.00	0.50	0.67	2
4	4	0.81	1.00	0.89	17
	5	0.00	0.00	0.00	3
	6	0.00	0.00	0.00	1
6	2	0.50	1.00	0.67	10
	3	1.00	0.18	0.31	11
	4	1.00	1.00	1.00	1
	5	1.00	1.00	1.00	1
	9	0.00	0.00	0.00	1
7	2	1.00	0.50	0.67	4
	3	0.67	1.00	0.80	8
	4	1.00	1.00	1.00	1
	5	0.00	0.00	0.00	2
8	1	0.00	0.00	0.00	1
	3	0.62	1.00	0.77	5
	4	0.60	1.00	0.75	6
	5	0.00	0.00	0.00	2
	6	0.00	0.00	0.00	3
	8	0.00	0.00	0.00	1
9	3	0.50	1.00	0.67	3
	5	0.00	0.00	0.00	2
	8	0.00	0.00	0.00	1

Tables 3 and 4 contain many missing classes or classes with very few observations, which prevented us from achieving good global quality metrics. We were not able to perform complete split for some of the classes because there was only one observation for them. The missing classes were due to the absence of patients with such characteristics in the Reference Hospital and not for an algorithmic reason. Table 5 summarizes the result of the model by eye and gaze.

**Table 5.** Global Accuracy, Weighted Precision, Weighted Recall, Weighted F1, and Validation Loss

Eye	Gaze	Accuracy	Precision	Recall	F1	Val_Loss
Left	1	0.45	0.33	0.38	0.29	1.6182
	2	0.76	0.38	0.50	0.43	0.9794
	3	0.67	0.77	0.67	0.50	0.9679
	4	0.81	0.66	0.81	0.72	1.0140
	6	0.58	0.75	0.58	0.50	0.9399
	7	0.73	0.69	0.73	0.67	0.0853
	8	0.61	0.37	0.61	0.46	2.2096
	9	0.50	0.25	0.50	0.33	1.4520
Right	1	0.42	0.28	0.42	0.31	1.5995
	2	0.71	0.63	0.71	0.65	0.8785
	3	0.56	0.80	0.56	0.49	1.4951
	4	0.73	0.66	0.73	0.37	1.1699
	6	0.54	0.50	0.54	0.49	1.0214
	7	0.92	0.84	0.92	0.88	0.5353
	8	0.57	0.60	0.57	0.46	0.9811

On the basis of the results, the model overfitted in cases of rare observations, sp the sample had to be increased. Gazes 1, 6, and 9 did not provide satisfactory results for the left eye, whereas gazes 1, 3, 6, and 8 did not provide satisfactory results for the right eye.

## Discussion

The mobile app developed herein for classification of ocular versions showed global accuracy ranging from 0.42 to 0.92 and precision ranging from 0.28 to 0.84. This accuracy range showed that the application had good potential for classification of eye versions, especially in some gazes like 2, 3, 4, 7, and 8 for the right eye and 2, 4, 7, and 8 for the left eye. Controlling the participants was difficult because the patients came from a university reference hospital that prioritizes the delivery of care to patients with specific pathologies, such as a Graves disease, which causes restrictive strabismus.

To develop the app, face positioning determined result accuracy. Kushner^22^ demonstrated that posture is important when examining eye motility. He developed a cervical range of motion device, an instrument designed to assess the range of motion of the cervical spine for accurate quantification of the magnitude of the patient's head abnormal posture, limitation of doubles, or range of single binocular vision in distance fixation. In our study, we used the facial landmark extractor from the Dlib library. This tool allowed us to locate the center of the face effectively and reduced the possibility of posture bias with face rotation.

Photo standardization was crucial because images with the same color saturation and brightness cannot always be obtained. This standardization also contributed to training the application where it did not consider variables other than eye position. If this step was not performed, all the photos in a certain position rated −2 could be brighter than the other ratings, and the application could consider this difference in brightness and not the eye position.

Table 2 lists the results regarding the accuracy of the application in classifying the gaze and eye version in a determined gaze. Such accuracy showed whether the application can standardize the eye versions. In addition, the practicality of using the application on smart phones confirmed its applicability in the ophthalmologist's routine.

Urist^16^ evaluated the versions by lateral reflection of light with limbus transillumination from illumination of the space between the eyes. According to this author, in 85% of normal eyes, reflexes located 10 mm from the limbus in the sclera of the abducted eye or 35° (Hirschberg scale) in the cornea of the adducted eye provide relevant evaluation in surgical cases, but they only discriminate between normal and abnormal muscle action.

Lim et al.^17^ evaluated version classification when excursion was quantified in degrees, to find that the accuracy of average difference between the observers was 0.2°, with 95% confidence limits of 2.6° and 3.1°. However, the authors described a possible selection bias because there were no strabismus patients in the study. Despite the high accuracy, the need for editing in Photoshop to identify the limbus was another disadvantage.

Other applications of artificial intelligence in strabismus have been reported. For example, Lu et al.^19^ described an automatic strabismus detection system for use in telemedicine. In their article, the authors depicted a set of tele-strabismus data established by ophthalmologists. Then, they proposed an end-to-end structure called Random Forest – Convolutional Neural Network (RF-CNN) to obtain automated detection of strabismus in the established set of tele-strabismus data. RF-CNN first segments the eye region in each individual image and further classifies the segmented eye regions with deep neural networks. The experimental results in the established strabismus dataset demonstrated that the proposed RF-CNN performs well in the automated detection of strabismus. In our application, the first step of ocular region detection and eye positioning followed the same proposal, with the difference that, on the basis of this information, we classified the eye version from the angle of eye excursion

Here, we respected the Classical Bioethical Principles applied in the use of artificial intelligence; nonmaleficence, beneficence, and justice are worth highlighting because the application could safely improve the quality of care, thereby improving patient's result.^20^^,^^21^ We also respected responsibility and respect for autonomy by ensuring the participant's responsibility and authorization through the informed consent. Besides justice and nonmaleficence, we compared this study to validated clinical studies that guarantee safety, effectiveness, and equity in the intervention.

The app presented in this study is an early prototype that is undergoing further development. Thus the app and preliminary evaluation studies presented here had some limitations. First, we were not able to use the application in individuals with facial deformities or corneal changes because it was difficult to recognize the 68 points with the facial landmark extractor. Another limiting factor was that, to avoid confusion bias due to possible conjunctival scars, patients that had already been operated could not be included. Further studies are suggested to broaden the spectrum of the use of the application, and studies that consider only the eye position and not variables such as facial, conjunctival, or corneal anomalies are recommended.

The app is a unique feature, and the differences between the app and the traditional semiological measure of ocular version were demonstrated. The app could complement ocular motility evaluation on the basis of objective classification of the ocular versions.

The application can be potentially used as an easy-to-apply tool to reduce time and increase diagnostic accuracy. However, exploratory research and validation are necessary.

## Supplementary Material

Supplement 1
